# The role of finerenone in the concomitant management of chronic kidney disease-type 2 diabetes and the implication for heart failure prevention and treatment

**DOI:** 10.1007/s10741-025-10520-3

**Published:** 2025-05-31

**Authors:** Pam R. Taub, Stephen J. Greene, Marat Fudim

**Affiliations:** 1 University of California San Diego, La Jolla, San Diego, CA USA; 2 School of Medicine, Duke University, Durham, NC USA; 3 Duke Clinical Research Institute, Durham, NC USA

**Keywords:** Heart failure, Finerenone, Chronic kidney disease, Mineralocorticoid receptor antagonist

## Abstract

The nonsteroidal mineralocorticoid receptor antagonist (MRA) finerenone is indicated in the United States for use in adults with chronic kidney disease (CKD) associated with type 2 diabetes (T2D). Results from the FIDELIO-DKD and FIGARO-DKD Phase 3 clinical trials showed a statistically significant reduction in the risk of CKD progression and cardiovascular events with finerenone versus placebo when added to maximally tolerated dose of an angiotensin-converting enzyme inhibitor or an angiotensin receptor blocker. The cardiovascular event risk reduction was primarily driven by the reduction in the risk of hospitalization for heart failure (HF). Recent results from the Phase 3 FINEARTS-HF trial in patients with HF with mildly reduced ejection fraction (HFmrEF) or HF with preserved ejection fraction (HFpEF) showed a significantly lower rate of a composite of total worsening HF events and death from cardiovascular causes with finerenone versus placebo. Further Phase 3 trials in additional HF populations are ongoing. The steroidal MRAs spironolactone and eplerenone are included in clinical practice guidelines for the treatment of symptomatic HF, but the highest class (grade 1) recommendations are in HF with reduced ejection fraction only. Based on the available evidence, finerenone presents as a new evidence-based therapy for HFpEF/HFmrEF in addition to its current application in CKD associated with T2D. The aim of our review article is to present the current evidence available on the potential kidney and cardioprotective effects of finerenone to inform healthcare professionals (particularly those who work in cardiology).

**Graphical Abstract:**

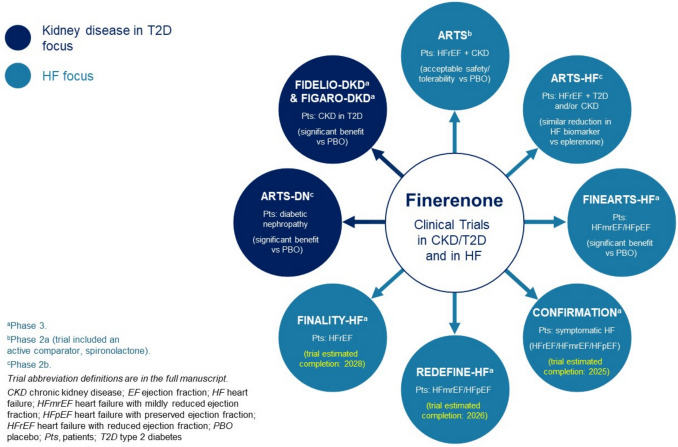

## Introduction

Cardiovascular–kidney–metabolic (CKM) syndrome represents the nexus of the pathophysiologic connections in cardiovascular disease, chronic kidney disease (CKD), and diabetes. Finerenone is a nonsteroidal mineralocorticoid receptor antagonist (ns-MRA) that has shown efficacy with acceptable tolerability in two Phase 3 clinical trials in adult patients with CKD associated with type 2 diabetes (T2D) and more recently in a Phase 3 trial involving patients with heart failure (HF) with mildly reduced or preserved ejection fraction. The aim of our review article is to present the current evidence available on the potential kidney and cardioprotective effects of finerenone to inform healthcare professionals (particularly those who work in cardiology).

## Burden of HF and CKD in the United States

HF is associated with significant morbidity and mortality. Results from an observational/longitudinal cohort study (EVOLUTION-HF) (n = 73,763 American adults) experiencing their first event of hospitalization for HF (HHF) showed that the most common reason for rehospitalization within 12 months of hospital discharge was another HF episode; the all-cause death (in-hospital) event rate for this group was 10.3 per 100 patient years [1].

In addition, the prevalence of CKD is also increasing in the United States. Currently, approximately 35.5 million American adults have CKD and approximately 808,000 Americans were living with end-stage kidney disease (ESKD) in 2020 [2]. Furthermore, globally, 42–53% of people with HF have comorbid CKD [3], representing a significant health and economic burden. For example, when comparing the annualized medical cost for comorbid CKD-HF vs. CKD only (based on 2016 or 2017 patient data from Kaiser Permanente Northwest), the annualized mean medical cost for comorbid CKD-HF is 2-to threefold higher than the cost for CKD only, even when accounting for patients with stage 5 (ESKD) CKD [4]. Thus, effective prevention and treatment strategies for both CKD and HF in the United States is of ongoing importance, both for improving individual quality of life and for reducing healthcare costs.

### A chronic inflammatory state in CKD and HF

There are several pathophysiologic mechanisms and risk factors that lead to the development of CKD and HF. Metabolic derangements that occur in diabetes and hypertension cause a prolonged inflammatory state [5]. This inflammatory state may cause endothelial cell dysfunction, hemodynamic dysregulation, oxidative stress, fibrosis, and vasoconstriction [6–9]. After initial compensatory responses, such as glomerular hyperfiltration in the kidneys, the heart and kidneys eventually lose structural and functional capacity. The filtration efficiency of the kidneys decreases that can be measured clinically as a reduction in the estimated glomerular filtration rate (eGFR) [10]. Structural kidney damage can be estimated clinically by assessment of albuminuria via the urine albumin-to-creatinine ratio (UACR) [10]. Some of the effects of kidney dysfunction include an increase in the volume of waste products in the blood, which can damage the heart, and hypertension due to sodium dysregulation [11, 12]. In the heart, the effects of sustained proinflammatory processes result in ventricular hypertrophy, fibrosis, and damage to the microvasculature supplying the heart, which may manifest as reduced cardiac output [11, 12]. As a result of reduced cardiac output, organs such as the kidneys and the liver may receive a reduced oxygen supply.

### Preventing CKD and HF

Given the significant public health and economic burden of CKD and HF, it is important to implement strategies to prevent both conditions. Common risk factors for CKD and HF include obesity, diabetes, and hypertension [13, 14]. Additionally, having CKD is a risk factor for developing HF, and having HF is a risk factor for CKD [12, 14]. Patients may also have albuminuria, which increases the risk of cardiovascular disease (including HF) and cardiovascular mortality [15]; and, a reduced eGFR, which increases a person’s risk of death, cardiovascular events, and hospitalization, independently of known risk factors [16].

Such bidirectional heart–kidney activity for incident disease is consistent with the physiologic interconnectivity of the heart and kidneys [11, 12]. Indeed, the term CKM syndrome is used to describe a multisystem disorder attributed to the intricate connections between obesity, diabetes, CKD, and cardiovascular disease, including HF [17]. CKM syndrome includes those at risk for cardiovascular disease and those with existing cardiovascular disease, and so not only highlights the interconnectivity of the heart and kidneys but also the significance of metabolic disorders such as diabetes as a factor in the development of and progression of CKD and HF [17].

The principal way of preventing the development of both HF and CKD is having a healthy lifestyle throughout the lifespan [13, 14, 18]. Prevention of CKD in individuals with diabetes includes maintaining a blood glucose A1C target below around 7%, and achieving optimal blood pressure (less than ~ 130/80 mmHg) as well as lipid targets [19, 20]. Maintenance of such targets may necessitate taking evidence-based medications, such as metformin (alone or in combination) for A1C, a statin for lipid control, and/or an ACEi or an ARB for blood pressure control [20, 21]. These preventative approaches also apply in the absence of diabetes in individuals who are still at risk of HF/CKD.

For those at continued risk of HF (stage A), or with pre-HF (stage B, asymptomatic), preventing development of symptomatic HF should be a priority. According to HF and CKD clinical practice guidelines, patients at risk for HF or CKD should be considered for natriuretic peptide-based biomarker screening for HF and UACR and eGFR screening for CKD [10, 22]. In addition, those with T2D and cardiovascular disease, or who are high risk for cardiovascular disease (placing them at risk for HF), should be considered for a sodium-glucose cotransporter 2 inhibitor (SGLT2i) to reduce their risk of developing HF (class 1 recommendation) [22]. Such patients should continue to adhere to lifestyle modifications and management strategies. A similar recommendation for SGLT2i in HF prevention is also included in European Society of Cardiology (ESC) guidelines (class/level 1 A), and these guidelines also include a recommendation for finerenone for patients with CKD and T2D to reduce their risk of HHF [23]. Asymptomatic individuals with structural and/or functional abnormalities (pre-HF, or stage B) should take an angiotensin-converting enzyme inhibitor (ACEi) (or an angiotensin receptor blocker [ARB] if ACEi intolerant and a recent myocardial infarction) plus a β-blocker if their left ventricular ejection fraction (LVEF) is ≤ 40%, and they should continue on lifestyle modifications and management strategies (class 1 recommendation) [22]. Final drug treatment choices for those at risk or with pre-HF depends on multiple factors, such as drug tolerability, comorbidities, contraindications, patient preference, and drug availability. Treatment recommendations for CKD are based on eGFR, UACR (or proteinuria), cardiovascular risk, and CKD progression risk [10]. As for HF prevention recommendations, lifestyle modifications and medication optimization for comorbid conditions are recommended for CKD [10].

### Symptomatic HF

The three main HF subtypes based on ejection fraction (EF) are HF with a reduced EF (HFrEF [LVEF, ≤ 40%]), HF with a mildly reduced EF (HFmrEF [LVEF, 41–49%]), and HF with a preserved EF (HFpEF [LVEF ≥ 50%]) [22]. Patients with an HFrEF diagnosis should continue with lifestyle modifications plus class 1 guideline-directed medical therapy recommendations consisting of an angiotensin receptor–neprilysin inhibitor (ARNi) or a renin–angiotensin–aldosterone system inhibitor (RAASi), a β-blocker, an MRA, an SGLT2-i, and a diuretic as needed, with dose adjustments and drug alternatives considered according to tolerability and contraindications [22].

HFpEF is increasing in prevalence (at least 50% of HF cases in the United States [24]) and is more common in older people, women, individuals with diabetes, those with obesity, and those with atrial fibrillation (AF), hypertension, and/or kidney dysfunction [25]. Compared with HFrEF, there are fewer classes of medical therapy definitely proven to improve outcomes in HFmrEF or HFpEF. SGLT2is are part of the foundational therapy for patients with HFpEF in the absence of absolute contraindications and have a class 2a–level recommendation by the AHA/American College of Cardiology (ACC)/Heart Failure Society of America (HFSA) for use in this HF subpopulation [22]. SGLT2is are also included as class 1 recommendations in the ESC guidelines (2023 focused update) for HFmrEF and HFpEF [23]. ARNi, MRAs and ARBs are also included in the AHA/ACC/HFSA recommendations for HFpEF but these are class 2b–level recommendations [22]. Blood pressure management and use of diuretics, as needed, are also strongly recommended by AHA/ACC/HSFA for the HFpEF population [22]. These recommendations for symptomatic HF do not stipulate what to do if a patient also has CKD, but the treatment approach used will likely consider factors such as pretreatment and ongoing kidney function, serum potassium, comorbidities, and current medications.

## Finerenone: an introduction

Finerenone is an ns-MRA approved by the United States Food and Drug Administration (FDA) in 2021 to reduce the risk of sustained eGFR decline, ESKD, cardiovascular death, nonfatal myocardial infarction (MI), and HHF in adults with CKD associated with T2D [26]. Hyperkalemia, which can cause arrythmias if severe, is a possible but uncommon side effect of taking finerenone and can be managed in most patients by monitoring of potassium levels and with dose adjustments [26]. Hyperkalemia rates are generally lower with finerenone than with steroidal MRAs, although there are limited head-to-head comparative trials [27, 28].

Finerenone is included in CKD-focused clinical practice guidelines, including the American Diabetes Association’s (ADA’s) Standards of Care in Diabetes and the Kidney Disease: Improving Global Outcomes (KDIGO) clinical practice guidelines for the management of CKD associated with T2D for individuals who are at risk of disease progression despite maximum tolerated dose of a RAASi [19, 29]. Finerenone is also included in these guidelines for cardiovascular risk reduction; it is recommended by the ESC for the prevention of HHF in patients with CKD associated with T2D [23].

**Fig. 1:**
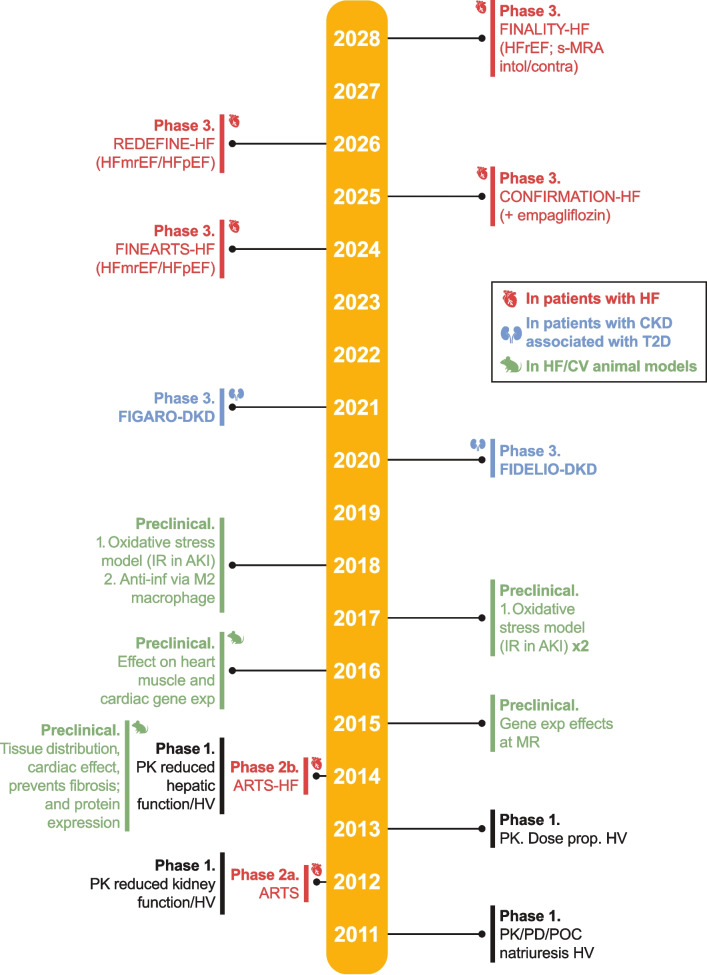
Timeline for finerenone-focused clinical trials in CKD/T2D and in HF. Trials are based on searches completed on clinicaltrials.gov on August 12, 2024. Japan and European/EU clinical trial registers were not searched. The year represents the year of study completion; for preclinical studies, the year represents the year the associated manuscript was published. AKI, acute kidney injury; CKD, chronic kidney disease; CV, cardiovascular; contra, contraindicated; HF, heart failure; HFmrEF, HF with mildly reduced ejection fraction; HFpEF, HF with preserved ejection fraction; HFrEF, HF with reduced ejection fraction; HV, healthy volunteer; IR, ischemia–reperfusion; untol, untolerated; MR, mineralocorticoid receptor; PD, pharmacodynamics; PK, pharmacokinetics; POC, proof of concept; s-MRA, steroidal mineralocorticoid receptor antagonist; T2D, type 2 diabetes

**Fig. 2:**
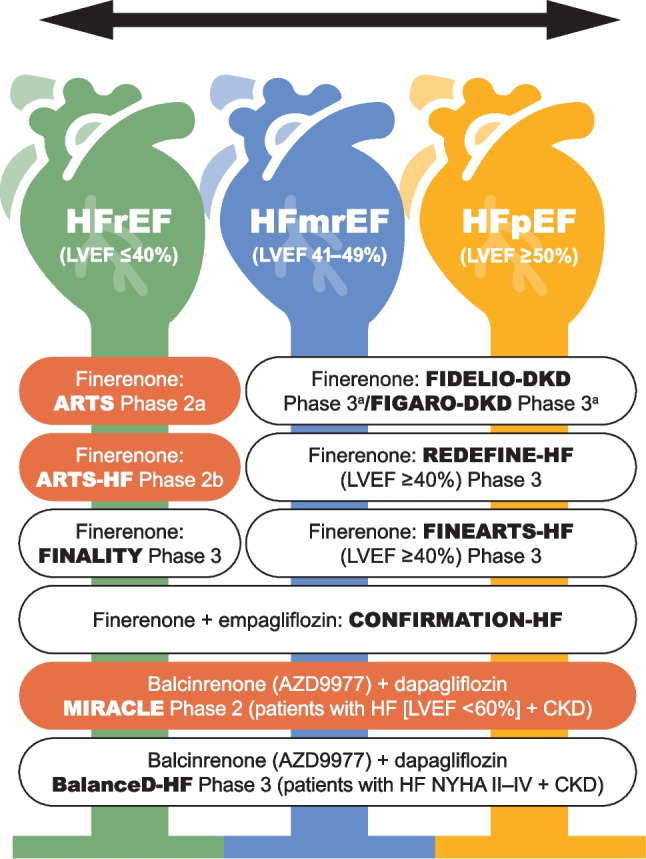
Nonsteroidal MRAs with trials in HF registered on clinicaltrials.gov website (searches performed on August 12, 2024) with completed, planned, or in-progress Phase 2 and/or Phase 3 interventional trials. Trials are presented by ejection fraction. Trials details in orange shade shape represent Phase 2 trials and trials in white shape represents Phase 3 trials. ^a^Not the primary patient population (in FIDELIO-DKD and FIGARO-DKD, the primary patient population, per main inclusion criteria, had CKD associated with T2D; patients with known HFrEF were excluded). CKD, chronic kidney disease; HF, heart failure; HFmrEF, HF with mildly reduced ejection fraction; HFpEF, HF with preserved ejection fraction; HFrEF, HF with reduced ejection fraction; LVEF, left ventricular ejection fraction; NYHA, New York Heart Association

The efficacy and safety of finerenone is also being investigated in Phase 3 trials in other therapy areas. Results from the recently completed FINEARTS-HF (NCT04435626) Phase 3 trial (Fig. 1, 2) demonstrated that finerenone significantly reduced the risk of the composite primary endpoint of total worsening HF events and death from cardiovascular causes vs. placebo in patients with symptomatic HFpEF or HFmrEF [30]. Finerenone is also being investigated in other symptomatic HF Phase 3 trials across the spectrum of HF subgroups (Fig. 1, 2).

## Comparing finerenone with steroidal MRAs

Steroidal MRAs include spironolactone and eplerenone and ns-MRAs include finerenone. Steroidal MRA uses include treatment of symptomatic HFrEF and treatment of resistant hypertension. Table 1 gives a summary of the results from Phase 3 trials of spironolactone, eplerenone, and finerenone in symptomatic HF [30–33].

**Table 1:** Summary of results from Phase 3 clinical trials in adult patients with chronic HF for the steroidal MRAs spironolactone and eplerenone and the ns-MRA finerenone

Finerenone	Spironolactone	Eplerenone
**Finerenone Trial to Investigate Efficacy and Safety Superior to Placebo in Patients with Heart Failure (FINEARTS-HF)** [30] Patients: • Symptomatic HF, LVEF ≥ 40% (defined as HFmrEF or HFpEF in the trial); evidence of structural heart disease and elevated natriuretic peptide levels Endpoints: • Primary: composite of total worsening HF events and death from CV causes • Secondary: change from baseline in KCCQ total symptom score; improvement in NYHA functional class; kidney composite outcome; death from any cause; first worsening HF event or death from CV causes Results: • Primary: 1083 primary-outcome events with finerenone vs. 1283 events with placebo (RR 0.84 [95% CI: 0.74–0.95; *p* = 0.007]) • Secondary: Least-squares mean change from baseline in KCCQ total score (difference vs. placebo 1.6 points; 95% CI: 0.8–2.3; *p* < 0.001) Improvement in NYHA functional class at month 12, 557 improvements with finerenone vs. 553 improvements with placebo (OR 1.01 [95% CI: 0.88–1.15]) Kidney composite: 75 events with finerenone vs. 55 events with placebo (HR 1.33 [95% CI: 0.94–1.89]) Death any cause: 491 with finerenone vs. 522 with placebo (HR 0.93 [95% CI: 0.83–1.06]) First worsening HF or death from CV causes: 624 with finerenone vs. 719 with placebo (HR 0.84 [95% CI: 0.76–0.94]) Adverse effects of interest: • Investigator-reported hyperkalemia higher with finerenone vs. placebo (9.7% vs. 4.2%); hypokalemia lower with finerenone vs. placebo • < 0.6% hyperkalemia events resulted in hospitalization (0.5% finerenone vs. 0.2% placebo) and none lead to death	**Randomized ALdactone Evaluation Study (RALES)** [31] Patients: • Severe HF; NYHA class III or IV HF with LVEF ≤ 35% (HFrEF); were taking an ACEi (if tolerated) and a loop diuretic Endpoints: • Primary: death from any cause • Secondary: death from cardiac causes, hospitalization for cardiac causes, the combined incidence of death from cardiac causes or hospitalization for cardiac causes, and a change in NYHA class Results: • Primary: 30% reduction in the risk of death from any cause with spironolactone vs. placebo (RR 0.70 [95% CI: 0.60–0.82; *p* < 0.001]) • Secondary: 32% reduction in the risk of combined endpoint of death from cardiac causes or hospitalization for cardiac causes with spironolactone vs. placebo (RR 0.68 [95% CI: 0.59–0.78; *p* < 0.001]) • NYHA class change: improved 41% (vs. 33% placebo); no change 21% (18% placebo); worsened in 38% (48% placebo); *p* < 0.001 Adverse effects of interest: • Serious hyperkalemia: 14 (2%) vs. 10 (1%) placebo • Gynecomastia in men: 55 (9%) vs. 8 (1%) placebo • Gynecomastia or breast pain in men: 61 (10%) vs. 9 (1%) placebo **Treatment of Preserved Cardiac Function Heart Failure with an Aldosterone Antagonist (TOPCAT)** [33] Patients: • At least 1 sign and at least 1 symptom of HF with LVEF ≥ 45% (defined as HFpEF in the trial); no requirement for certain baseline medications in inclusion criteria Endpoints: • Primary: composite of death from CV causes, aborted cardiac arrest, or hospitalization for management of HF • Secondary: death from any cause, hospitalization for any reason, MI, stroke Results: • Primary outcome occurred in 320 (18.6%) with spironolactone vs. 351 (20.4%) with placebo (HR 0.89 [95% CI: 0.77–1.04; *p* = 0.14]) • Secondary: no significant differences with spironolactone vs. placebo (*p* > 0.05) Adverse effects of interest: • Higher hyperkalemia with spironolactone vs. placebo (18.7% vs. 9.1%) but lower hypokalemia (16.2% vs. 22.9%) • Discontinuation of study drug due to breast tenderness or gynecomastia significantly more frequent with spironolactone vs. placebo	**Eplerenone Post–acute myocardial infarction Heart failure Efficacy and SUrvival Study (EPHESUS)** [32] Patients: • Acute MI 3–14 days prior to randomization; HF with LVEF ≤ 40% (HFrEF); symptomatic HF unless having diabetes (but still meet criteria stated above); taking SOC (including ACEis, ARBs, diuretics) Endpoints: • Coprimary: time to death from any cause *and* time to death from CV causes or first hospitalization for a CV event, including HF, recurrent acute MI, stroke, or ventricular arrhythmia • Secondary: includes death from CV causes and death from any cause or any hospitalization Results: • Primary: 15% reduction in risk of death from any cause (RR 0.85 [95% CI: 0.75–0.96; *p* = 0.008]); 13% reduction in risk of death from CV causes or hospitalization for CV events vs. placebo (RR 0.87 [95% CI: 0.79–0.95; *p* = 0.002]) • Secondary: 8% reduction in risk of death from any cause or any hospitalization vs. placebo (RR 0.92 [95% CI: 0.86–0.98; *p* = 0.02]) Adverse effects of interest: • Serious hyperkalemia (≥ 6 mmol/L): 180 (5.5%) vs. 126 (3.9%) placebo (*p* = 0.002) • Gynecomastia in men: 12 (0.5%) vs. 14 (0.6%) placebo (*p* = NSD) • 0.06 mg/dL (5.3 µmol/L) increase serum creatinine concentration (1 year) vs. 0.02 mg/dL with placebo (*p* < 0.001)

*ACEi* angiotensin-converting enzyme inhibitor, *ARB* angiotensin receptor blocker, *CV* cardiovascular, *HF* heart failure, *HFmrEF* heart failure with mildly reduced ejection fraction, *HFpEF* heart failure with preserved ejection fraction, *HFrEF* heart failure with reduced ejection fraction, *HHF* hospitalization for heart failure, *HR* hazard ratio, *KCCQ* Kansas City Cardiomyopathy Questionnaire, *LVEF* left ventricular ejection fraction, *MI* myocardial infarction; *NSD* no significant difference; *NYHA* New York Heart Association, *OR* odds ratio, *RR* relative risk; *SOC* standard of care

Both steroidal MRAs and ns-MRAs block binding of aldosterone to the mineralocorticoid receptor (MR) [34]. However, the potency, selectivity, and distribution pattern of steroidal MRAs and finerenone is different. Finerenone has higher antagonistic (inhibitory) potency at the MR [35] and inverse agonism compared with eplerenone and is more selective in its effects than spironolactone. Additionally, in the absence of aldosterone, finerenone blocked recruitment of coactivators (cofactors are needed for successful nuclear translocation and receptor-mediated end effects) [35]. Finerenone’s greater potency compared with eplerenone may be due to the bulky antagonist (inverse agonist) nature of the drug. This is absent in steroidal MRAs, with finerenone causing a protrusion from the binding pocket of the MR (making it highly selective) that blocks cofactor recruitment to the MR [35–37]. Overall, finerenone impairs transcriptional activation of MR target genes [38]. Both finerenone and eplerenone have high selectivity for the MR but are weakly selective for non-MR (androgen) receptors (AR); additionally, finerenone has higher selectivity for the MR than eplerenone (~ 160-fold more selective for MR vs. eplerenone, ~ 21-fold more selective) [36]. Spironolactone has low selectivity for the MR vs. AR (~ threefold more selective for the MR vs. AR), which means it has greater potential to block ARs, which is associated with sexual side effects such as gynecomastia, especially at higher doses [36, 39–41].

Finerenone is equally distributed (approximately 1:1) in the heart and kidneys [42]. In contrast, spironolactone and eplerenone are distributed at a much higher concentration in the kidneys than in the heart [42–44]. Such distribution pattern differences may help account for heart and kidney effects of finerenone and the greater potassium-sparing (diuretic), antihypertensive effects (via tubular action) of the steroidal MRAs [26, 41, 45].

Hyperkalemia can occur if significant kidney dysfunction is present but is also a possible side effect of treatment with any available MRA. Patients taking a steroidal MRA and who are at high risk of hyperkalemia may be advised to reduce their dietary consumption of potassium and also consider taking a potassium-binding drug, such as patiromer [46, 47], or sodium cyclosilicate. Separate Phase 3 clinical trials with spironolactone, eplerenone, and finerenone have shown higher hyperkalemia rates among participants taking one of these MRAs vs. those taking placebo [31, 32, 48, 49]. Participants taking part in these trials are already at high baseline hyperkalemia risk due to existing kidney dysfunction and/or have above-normal potassium levels. Hyperkalemia can be a reason for discontinuing MRA drug treatment. However, results from two Phase 2 trials (described in more detail in a later section) of finerenone vs. steroidal MRA effects, showed a higher occurrence of hyperkalemia or serum potassium increases from baseline with eplerenone or spironolactone compared with finerenone (mean change from baseline to day 90 in serum potassium, + 0.262 mmol/L with eplerenone [target, 50 mg/day] vs. + 0.119–0.202 mmol/L in each of the finerenone dose groups [2.5 to 5, 5 to 10, 7.5 to 15, 10 to 20, and 15 to 20 mg/day] [27]; and 7 hyperkalemia events with spironolactone [25 or 50 mg/day] vs. 1–3 events across the finerenone 2.5, 5, and 10 mg/day dose groups [28]). The reasons for this include the weaker potassium-sparing effect of finerenone and its shorter half-life (2–3 h) and absence of active metabolites vs. spironolactone’s half-life of 1.4 h + active metabolites (half-life 13.8–16.5 h); and vs. eplerenone half-life of 4–6 h + no active metabolites [26, 41, 45]. The product information labels for these MRAs recommend monitoring of serum potassium levels during treatment, and, if hyperkalemia is diagnosed, dose reduction, pausing dosing, or stopping the drug as needed is recommended; for finerenone, potassium monitoring is recommended and dosing paused if potassium is > 5.5 mEq/L and dosing restated once the hyperkalemia has been treated [26]. Similar response recommendations are present for eplerenone [45]; for spironolactone, the recommendation is to reduce the dose or discontinue treatment completely [41].

## Finerenone clinical trials

Figure 1 shows the timeline for the main finerenone preclinical studies and Phase 2–3 clinical trials in CKD associated with T2D and in HF.

### Finerenone in patients with CKD associated with T2D

Finerenone’s current indication in CKD associated with T2D is based on results from 2 large placebo-controlled Phase 3 trials called FIDELIO-DKD (Finerenone in Reducing Kidney Failure and Disease Progression in Diabetic Kidney Disease) and FIGARO-DKD (Finerenone in Reducing Cardiovascular Mortality and Morbidity in Diabetic Kidney Disease). Table 2 shows the key inclusion criteria and results from these trials and also for the Phase 2 ARTS-DN trial [48–51]. After the Phase 3 trials had completed, a prespecified pooled analysis called FIDELITY (FInerenone in chronic kiDney diseasE and type 2 diabetes: Combined FIDELIO-DKD and FIGARO-DKD Trial programme analYsis), which combined data from both of these trials (n = 13,026 [51]), was completed.

**Table 2:** Overview of study design and key results from the finerenone Phase 2 ARTS-DN trial and Phase 3 FIDELIO-DKD and FIGARO-DKD trials (and FIDELITY pooled analysis) in patients with CKD associated with T2D

	**ARTS-DN** [50]	**FIDELIO-DKD** [48]	**FIGARO-DKD** [49]	**FIDELITY prespecified pooled analysis** [51]
Main inclusion criteria	• T2D diagnosis • UACR ≥ 30 mg/g (target, ≥ 35% with UACR ≥ 300 mg/g) and eGFR > 30 mL/min/1.73 m^2^ • Taking at least minimum recommended dosage of RAASi • Serum potassium ≤ 4.8 mmol/L	• Adults, T2D diagnosis, CKD diagnosis treated with maximum dose ACEi or ARB (RAASis); serum potassium ≤ 4.8 mmol/L *CKD defined as:* • UACR 30 to < 300 mg/g and eGFR 25 to < 60 mL/min/1.73 m^2^ + history of diabetic retinopathy *OR* • UACR 300 to 5000 mg/g and eGFR 25 to < 75 mL/min/1.73 m^2^	• Adults, T2D diagnosis, CKD diagnosis treated with maximum dose ACEi or ARB (RAASi); serum potassium ≤ 4.8 mmol/L *CKD defined as:* • UACR 30 to < 300 mg/g and eGFR 25 to 90 mL/min/1.73 m^2^ *OR* • UACR 300 to 5000 mg/g and eGFR ≥ 60 mL/min/1.73 m^2^	• See FIDELIO-DKD and FIGARO-DKD
Treatments	• Finerenone (1.25, 2.5, 5, 7.5, 10, 15, 20 mg) + RAASi • Placebo + RAASi	• Finerenone (10 mg; target 20 mg) + RAASi • Placebo + RAASi	• Finerenone (10 mg; target 20 mg) + RAASi • Placebo + RAASi	• See FIDELIO-DKD and FIGARO-DKD
Primary and secondary endpoints/outcomes	• Primary: ratio of UACR at day 90 vs. at baseline • Other variables included safety, decreases in eGFR, (≥ 30%, ≥ 40%, ≥ 57%), change in UACR day 30 and 60 vs. baseline	• Primary: composite of kidney failure, sustained decrease of at least 40% in eGFR from baseline over ≥ 4 weeks, or death from renal causes (time-event analysis) • Key secondary: composite of death from CV causes, nonfatal MI, nonfatal stroke, or HHF (time-event analysis)	• Primary: composite of death from CV causes, nonfatal MI, nonfatal stroke, or HHF (time–event analysis) • Key secondary: composite of first occurrence of kidney failure, a sustained decrease from baseline of ≥ 40% in eGFR for ≥ 4 weeks, or death from renal causes	• Composite CV outcome of time to CV death, nonfatal MI, nonfatal stroke, or HHF, *and* a composite kidney outcome of time to first onset of kidney failure, sustained ≥ 57% decrease in eGFR from baseline over ≥ 4 weeks, or kidney death
Key results	• Mean placebo corrected ratio of UACR at day 90 vs. baseline; significant decrease in UACR with 7.5, 10, 15, and 20 mg finerenone groups (*p* < 0.005) • No effect on eGFR vs. placebo	• Occurrence of the primary outcome event significantly lower with finerenone vs. placebo (17.8% finerenone vs. 21.1% placebo; HR 0.82 [95% CI: 0.73–0.93; *p* = 0.001]) • Occurrence of the secondary outcome event significantly lower with finerenone vs. placebo (13.0% finerenone vs. 14.8% placebo; HR 0.86 [95% CI: 0.75–0.99; *p* = 0.03])	• Occurrence of the primary outcome event significantly lower with finerenone vs. placebo (12.4% finerenone vs. 14.2% placebo; HR 0.87 [95% CI: 0.76–0.98; *p* = 0.03], driven by HHF results) • No significant difference in occurrence of the secondary outcome vs. placebo (9.5% finerenone vs. 10.8% placebo; HR 0.87 [95% CI: 0.76–1.01])	• Composite CV outcome occurred in 12.7% finerenone and 14.4% placebo (HR 0.86 [95% CI: 0.78–0.95; *p* = 0.0018]), driven by HHF results (*p* = 0.0030 vs. placebo) • Composite kidney outcome significantly lower with finerenone: 5.5% vs. 7.1% placebo (HR 0.77 [95% CI: 0.67–0.88; *p* = 0.0002])
Adverse effects of special interest	• 1.5% increase in potassium to at least 5.6 = discontinuation treatment	• More hyperkalemia events related to trial regimen with finerenone vs. placebo (11.8% vs. 4.8%) and more discontinuations of treatment with finerenone (2.3% vs. 0.9%); no fatal hyperkalemia cases	• More hyperkalemia events related to trial regimen with finerenone vs. placebo (6.5% vs. 3.1%) and more discontinuations of treatment with finerenone (1.2% vs. 0.4%)	• Hyperkalemia-related events occurred more frequently with finerenone (14.0%) vs. placebo (6.9%), but no hyperkalemia-related events were fatal and only a small proportion led to permanent treatment discontinuation (1.7% [incidence rate 0.66 per 100 patient-years] and 0.6% [incidence rate 0.22 per 100 patient-years], respectively) or hospitalization (0.9% and 0.2%, respectively)
Overall conclusions	In patients with diabetic nephropathy taking a RAASi, finerenone improved UACR relative to placebo	In patients with CKD and T2D, finerenone was associated with lower risks of CKD progression and CV events vs. placebo	In patients with CKD associated with T2D, finerenone significantly improved CV outcomes vs. placebo	Across the spectrum of patients with CKD associated with T2D, finerenone reduced the risk of clinically important CV and kidney outcomes vs. placebo

*ACEi* angiotensin-converting enzyme inhibitor, *ARB* angiotensin receptor blocker, *CKD* chronic kidney disease, *CV* cardiovascular, *eGFR* estimated glomerular filtration rate, *HHF* hospitalization for heart failure, *HR* hazard ratio, *MI* myocardial infarction, *RAASi* renin–angiotensin–aldosterone system inhibitor, *T2D* type 2 diabetes, *UACR* urine albumin-to-creatinine ratio

These trials and subsequent FIDELITY analysis demonstrated greater reduction in the risk of clinically important cardiovascular and kidney outcomes with finerenone (plus maximum tolerated dose of a RAASi) vs. placebo across the spectrum of CKD patients with T2D. In both trials, the effect of finerenone on HF in patients with CKD and T2D was assessed via the HHF outcome for finerenone vs. placebo. The cardiorenal effects of finerenone relative to placebo in the Phase 3 trials were observed irrespective of concomitant SGLT2i use [52].

### Reduction in risk of hospitalization for HF with finerenone

In FIDELIO-DKD and FIGARO-DKD, patients with symptomatic chronic HFrEF and persistent symptoms (New York Heart Association [NYHA] class II–IV) were excluded from the studies [48, 49]. However, patients with HF with stage B asymptomatic HFrEF or HFpEF or stage C HF (i.e., current or previous signs and symptoms of HFmrEF or HFpEF) could be included (Fig. 2) [53, 54].

HHF was included as part of the primary (cardiovascular) composite (time to event analysis) outcome in the FIGARO-DKD trial [49]. The primary composite outcome was death from cardiovascular causes, nonfatal MI, nonfatal stroke, or HHF. The incidence of HHF was lower with finerenone (117 events [3.2%]) vs. placebo (163 events [4.4%]), and finerenone was associated with a significantly lower risk of the primary cardiovascular composite outcome vs. placebo (*p* = 0.03). This reduction in the risk of HHF was the main component that drove the efficacy of finerenone on the primary (cardiovascular) outcome in the trial. In FIDELIO-DKD, HHF was included as part of the secondary (cardiovascular) composite (time to event analysis) outcome [48]. The secondary composite outcome was death from cardiovascular causes, nonfatal MI, nonfatal stroke, or HHF. The incidence of HHF was lower with finerenone (139 [4.9%] events) vs. placebo (162 [5.7%] events), although the 95% CI crosses 1 (95% CI: 0.68–1.08); overall, finerenone was associated with a significantly lower risk of the secondary (cardiovascular) composite outcome vs. placebo (*p* = 0.03).

In the FIDELITY analysis, HHF was included as part of the (cardiovascular) composite outcome of time to cardiovascular death, nonfatal MI, nonfatal stroke, or HHF [51]. The incidence of HHF was significantly lower with finerenone vs. placebo (256 vs. 325 events; HR 0.78 [95% CI: 0.66–0.92; *p* = 0.0030]), and overall, finerenone was associated with a significantly lower risk of the cardiovascular composite outcome vs. placebo. This finding is consistent with the ESC Class/level 1 A guideline recommendation to consider finerenone to prevent HHF among patients with T2D and CKD.

### Subgroup of patients with HF in FIDELIO-DKD and FIGARO-DKD

Prespecified analyses from both the FIGARO-DKD and FIDELIO-DKD clinical trials included focusing on the patients with a history of HF at baseline. In the FIGARO-DKD trial, 571 (7.8%) patients had a history of HF at baseline [53]. Similarly, in FIDELIO-DKD, 436 (7.7%) patients had a history of HF [54]. In the FIDELIO-DKD trial, history of HF did not modify the effect of finerenone vs. placebo on the components of the composite cardiovascular outcome, including HHF (HR 0.65 [95% CI: 0.39–1.09] and HR 0.95 [95% CI: 0.73–1.22] for patients with or without HF, respectively [interaction *p* = 0.20]); this was also the case for kidney outcomes [54]. Similarly, the effects of finerenone on improving HF outcomes observed in the FIGARO-DKD clinical trial were not modified by a history of HF [53].

The prespecified FIDELITY analysis combined data from the FIDELIO-DKD and FIGARO-DKD clinical trials, providing a larger sample size. Results from a subgroup analysis from FIDELITY [55] showed that finerenone reduced the risk of HF-related outcomes in patients with T2D across a broad spectrum of CKD stages. These HF-results were consistent across both trials suggesting that earlier intervention in the CKD disease course is likely to provide the greatest long-term benefit on HF-related outcomes. Furthermore, these results precede the recent completion of the FINEARTS-HF trial that also provides finerenone as a potential treatment for established HF. The treatment benefits of finerenone in FIGARO-DKD and FIDELIO-DKD were not modified by baseline eGFR and UACR categories; however, the magnitude of the treatment benefit tended to favor patients with less advanced CKD (i.e., patients with a higher baseline eGFR [≥ 60] or lower baseline UACR [< 300 mg/g]).

## Finerenone in patients with symptomatic HF: phase 2 trials

Finerenone’s effect in patients with HF and CKD was tested in Phase 2 clinical trials (ARTS and ARTS-HF). ARTS (minerAlocorticoid Receptor antagonist Tolerability Study) (NCT01345656) was a Phase 2a clinical trial in patients with HFrEF (NYHA class II–III symptoms), normal serum potassium, plus mild/moderate CKD [28]. It was carried out in 2 parts; Part A was placebo controlled and involved 65 patients with HFrEF plus mild CKD (finerenone was tested at 2.5, 5, and 10 mg/day). The main focus of Part A was to determine the safety and tolerability of finerenone in this group of patients relative to placebo. After Part A had completed, the trial moved to Part B (the safety and tolerability of finerenone was considered acceptable by an independent data monitoring committee). Part B involved 392 patients diagnosed with HFrEF plus moderate CKD (mean eGFR, 47 mL/min/1.73 m^2^) and was placebo controlled with active spironolactone comparator (finerenone was tested at 2.5, 5, or 10 mg/day [once daily] or 10 mg/day [5 mg twice daily] and spironolactone at an initial dose of 25 mg/day and uptitrated to 50 mg/day). The primary endpoint for Part B was change in serum potassium with finerenone; the proportion of hyperkalemia cases was higher with finerenone than with placebo but was lower than spironolactone (specific values provided in an earlier section), and most treatment-emergent adverse events with finerenone were mild.

The ARTS-HF (minerAlocorticoid Receptor antagonist Tolerability Study-Heart Failure) (NCT01807221) trial involved patients with HFrEF with worsening chronic features requiring hospitalization and treatment with intravenous diuretics [27]. The patients in this trial also had T2D and/or CKD (eGFR > 30 if with T2D or eGFR 30–60 without T2D). The effects of finerenone (n = 834 across 5 doses) were compared with eplerenone (n = 221) active comparator (there was no placebo control). In this trial, finerenone was tested across 5 dose groups (2.5 to 5, 5 to 10, 7.5 to 15, 10 to 20, and 15 to 20 mg/day) and eplerenone at a starting dose of 25 mg/2 days, then 25 mg/day, to a final dose of 50 mg/day. The primary efficacy endpoint was an N-terminal pro-B-type natriuretic peptide level decrease of > 30% to day 90 vs. baseline; this was similar for finerenone vs. eplerenone. Incidences of the exploratory composite endpoints (death from any cause, cardiovascular hospitalization, or emergency presentation for worsening HF until day 90) were lower with finerenone (at doses above 2.5–5.0 mg once daily) vs. eplerenone (25–35 events with finerenone vs. 51 events with eplerenone). The mean change from baseline to day 90 in serum potassium concentration was greater with eplerenone vs. finerenone (specific values provided in an earlier section), but reported hyperkalemia incidence was similar.

## Finerenone in patients with symptomatic heart failure: phase 3 trials

There are 4 Phase 3 clinical trials testing the effect of finerenone in patients with HF. Three are ongoing and 1 (FINEARTS-HF [NCT04435626]) has recently completed (Fig. 1; Table 1). CONFIRMATION-HF (NCT06024746), REDEFINE-HF (NCT06008197), and FINALITY-HF (NCT06033950) are ongoing, with anticipated study completion years of 2025, 2026, and 2028, respectively (Fig. 1).

In the FINEARTS-HF Phase 3 trial, 6001 adult patients with symptomatic HF, an LVEF of ≥ 40% (HFmrEF or HFpEF), plus evidence of structural heart disease and elevated levels of natriuretic peptides were randomized to receive finerenone (*n* = 3003) or placebo (*n* = 2998) [30]. A large proportion of patients were taking a loop diuretic (~ 87%) and/or a β-blocker (~ 85%) at baseline. The primary outcome was a composite of total worsening HF events and death from cardiovascular causes; the number of outcome events was significantly less with finerenone vs. placebo (1083 versus 1283; rate ratio [RR] 0.84, 95% CI 0.74–0.95; *p* = 0.007). If the individual components of the primary composite endpoint are reviewed, total worsening HF events were significantly lower with finerenone versus placebo (RR 0.82 [95% CI 0.71–0.94]; *p* = 0.006) but there was no significant difference versus placebo in death from cardiovascular causes (RR 0.93 95% CI 0.78–1.11).

For the secondary endpoint analysis, finerenone was associated with a significant improvement in patient-reported health status (least squares mean Kansas City Cardiomyopathy Questionnaire score difference, 1.6 points [95% CI: 0.8–2.3]) but not with the other secondary endpoints, which included improvement in NYHA function class at 12 months and the kidney composite outcome of sustained decrease in the eGFR of ≥ 50%, a sustained decline in the eGFR to < 15 mL/min/1.73 m^2^, or the initiation of long-term dialysis or kidney transplantation, assessed in a time-to-event analysis (95% CIs crossed 1, meaning nonsignificant result). More cases of hyperkalemia were reported with finerenone vs. placebo (289 [9.7%] vs. 125 [4.2%]); however, hospitalization for hyperkalemia was rare (0.5% with finerenone vs. 0.2% with placebo), and no cases of hyperkalemia led to death. Finerenone was also associated with fewer total events of hypokalemia vs. placebo (defined in the trial as serum potassium < 3.5 mmol/liter); 145 (5.0%) vs. 299 (10.3%), respectively.

## Other nonsteroidal MRAs in clinical development: CKD or HF

**Fig. 3:**
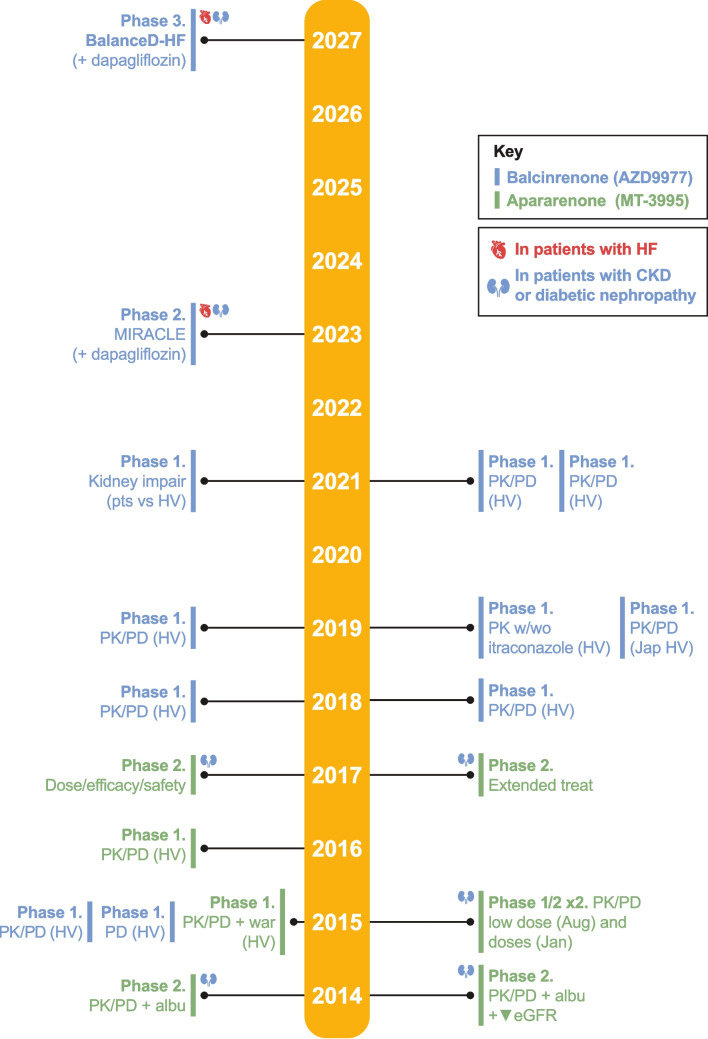
Timeline for clinical development of the ns-MRA balcinrenone (AZD9977) and apararenone (MT-3995)***.*** Trials are based on searches completed on clinicaltrials.gov on August 12, 2024. Japan and European/EU clinical trial registers were not searched. “Year” in the figure represents year of study completion. albu, albuminuria; CKD, chronic kidney disease; eGFR, estimated glomerular filtration rate; HF, heart failure; HV, healthy volunteer; impair, impairment; Jap, Japanese; PD, pharmacodynamics; PK, pharmacokinetics; pts, patients; war, warfarin

There are 2 other ns-MRAs with Phase 2/3 trials registered on clinical trials.gov with clinical development programs in progress: apararenone (MT-3995) for diabetic nephropathy and balcinrenone (AZD9977; Fig. 2) for HF, [56–59]. Figure 3 shows the phase 1–3 clinical trial development timeline for these two ns-MRAs.

## Discussion and summary

Advanced-stage CKD and microalbuminuria are associated with an increased risk of developing cardiovascular disease, including HF. In addition, chronic HF increases a person’s risk of developing CKD due to a reduced oxygen supply to the kidneys caused by the fall in cardiac output. Individuals with poorly controlled hypertension or diabetes plus preexisting cardiovascular risk factors are at an even higher risk of developing CKD and HF. CKM syndrome is used to describe the cumulation of the pathophysiologic connections in cardiovascular disease, CKD, and diabetes.

Our review article focused on the ns-MRA finerenone. Finerenone is indicated for use in adults with CKD associated with T2D. However, more recently, results from the FINEARTS-HF trial (patients with HFmrEF and HFpEF, with or without T2D) showed that finerenone has heart and cardiovascular-beneficial effects in HF, as well greater improvements in patient-reported outcomes vs. placebo. Such kidney- and cardiovascular-protective effects of finerenone likely originate from the antifibrotic and anti-inflammatory events that precede binding of finerenone to the MR.

The steroidal MRAs spironolactone and eplerenone are alternatives to ns-MRAs and are indicated for use in conditions including HFrEF, but neither of these drugs have been proven effective in dedicated trials of CKD populations. There are ongoing Phase 3 trials testing the effect of finerenone across the spectrum of ejection fraction, as well an ongoing Phase 3 trial testing the effect of spironolactone in HFpEF (SPIRRIT; NCT02901184); thus, it will be of interest to learn the outcomes of similar analyses in the future when more data are available.

In summary, finerenone in combination with the maximum tolerated dose of a RAASi provides an effective evidence-based treatment option for patients living with CKD associated with T2D. In addition, results from Phase 3 clinical trials also support finerenone’s efficacy in reducing the risk of HHF in patients with CKD/T2D and in reducing the risk of worsening of HF in patients living with HFmrEF or HFpEF; as such, it should be considered by healthcare professionals for both its potential kidney- and cardiovascular-protective effects.

## Data Availability

No datasets were generated or analysed during the current study.
